# The Global Prevalence of Antibiotic Resistance and Shiga Toxin-Producing *Escherichia coli* in Chickens: A Systematic Review and Meta-Analysis (2011–2024)

**DOI:** 10.3390/antibiotics14060568

**Published:** 2025-05-31

**Authors:** Tsepo Ramatla, Nkhebenyane Jane, Mohapi Dineo, Tawana Mpho, Motlhaoloa Tshegofatso, Ntelekwane George Khasapane

**Affiliations:** 1 Centre for Applied Food Safety and Biotechnology, Department of Life Sciences, Central University of Technology, 1 Park Road, Bloemfontein 9300, South Africa; snkheben@cut.ac.za (N.J.); dineoattela@gmail.com (M.D.); tk.motlhaoloa@gmail.com (M.T.); nkhasapane@cut.ac.za (N.G.K.); 2 Department of Zoology and Entomology, University of the Free State, Private Bag x13, Phuthaditjhaba 9866, South Africa; 2027028865@ufs4life.ac.za

**Keywords:** Shiga toxin-producing *Escherichia coli* (STEC), chicken, serotypes, antibiotic resistance

## Abstract

**Background:** Shiga toxin-producing *E. coli* (STEC) are important foodborne pathogens that cause serious public health consequences worldwide. This study conducted a systematic review and meta-analysis of the global prevalence of antibiotic resistance and STEC in chickens. **Methods:** The assessment of previous study records was carried out following the Preferred Reporting Items for Systematic Reviews and Meta-Analyses (PRISMA) guidelines. Heterogeneity between studies was assessed using Cochrane’s Q test and I^2^ test statistics based on the random effects model, and comprehensive meta-analysis (CMA) software v4.0 was used to analyse the pooled prevalence estimate (PPE) of antibiotic resistance and STEC in chickens. **Results:** A total of 61 studies comprising 823 STEC from 18 countries were included in this study. The overall pooled prevalence of STEC was 8.9% (95% CI: 0.620–0.126). m-PCR assay showed the highest PPE of 21.0% (95%: 0.088–0.420). *stx1* had the higher PPE of 12.9% (95%: 0.081–0.199), while *stx2* had a PPE of 11.8% (95%: 0.077–0.176). Furthermore, the serotype O157 had the higher PPE of 80.5% (95%: 0.520–0.940). The isolates were resistant to the following antibiotics: amoxicillin and clavulanic acid, chloramphenicol, tetracycline, ciprofloxacin, gentamycin, ampicillin, neomycin, and amoxicillin. **Conclusions:** These findings may assist in the prevention and control of STEC in chickens globally. To minimise the spread of STEC and antibiotic resistance, future foodborne pathogen prevention and control programmes should prioritise increasing laboratory capacity for the early identification of antibiotic resistance.

## 1. Introduction

*Escherichia coli* is a Gram-negative, rod-shaped, flagellated, facultatively anaerobic, non-sporulating bacterium that belongs to the *Enterobacteriaceae* family [1]. The six groups or pathotypes of *E. coli* strains are likely the result of the spread of coding plasmids carrying pathogenicity genes from other species, which gave rise to strains capable of causing severe illnesses. These include Enteropathogenic *E. coli* (EPEC), Enteroaggregative *E. coli* (EAEC), Enterotoxigenic (ETEC), Adherent-Invasive (AIEC), Diffusely adherent *E. coli* (DAEC), Avian pathogenic (APEC), and Enterohemorrhagic *E. coli* (EHEC) [2,3]. Each *E. coli* pathotype has different pathogenic processes and a diverse set of virulence proteins encoded by discrete gene clusters [3,4]. The majority of these pathotypes are foodborne pathogens that pose a threat to public health and have been responsible for multiple deadly outbreaks in both developed and developing countries [5].

Shiga toxin-producing *Escherichia coli* (STEC) is one of the most important zoonotic pathogens in the food supply chain, causing gastrointestinal issues in humans throughout the world [6,7]. STEC is distinguished by its ability to produce Shiga toxins (*Stx1* and/or *Stx2*), which are powerful cytotoxins that induce severe disease, including bloody diarrhoea and haemolytic uremic syndrome [8]. The amino acid sequences of the two primary families of Shiga toxins (*Stx*), *Stx1* and *Stx2*, are 70% identical [7,9].

Due to the ongoing reduction in the discovery and development of new antibiotics, the spread of antibiotic resistance (ABR) continues to be a global public health concern and places a heavy financial burden on the health sector [10]. Shiga toxin-producing *E. coli* displays antibiotic resistance by intrinsic or acquired mechanisms from other bacteria [11,12]. When AMR genes are present in mobile genetic elements, they increase the risk of AMR spreading among STEC bacteria and other bacteria, thereby limiting the available therapeutic choices for humans [11,12].

Several systematic reviews have investigated STEC in various contexts, including STEC in bovine meat and meat products in Brazil [13], cattle in China [14], transmission pathways [15], non-O157 STEC serogroups in global cattle carcasses [16], and the presence of *Escherichia coli* O157:H7 in Africa from a “One Health” perspective [17]. There is limited information on the comprehensive data needed to estimate the global prevalence of antibiotic resistance and STEC in chickens. The current study is a systematic review and meta-analysis aimed at providing a comprehensive prevalence of antibiotic resistance and STEC in chickens based on available data published globally.

## 2. Results

### 2.1. Study Selection and Identification

**Figure 1:**
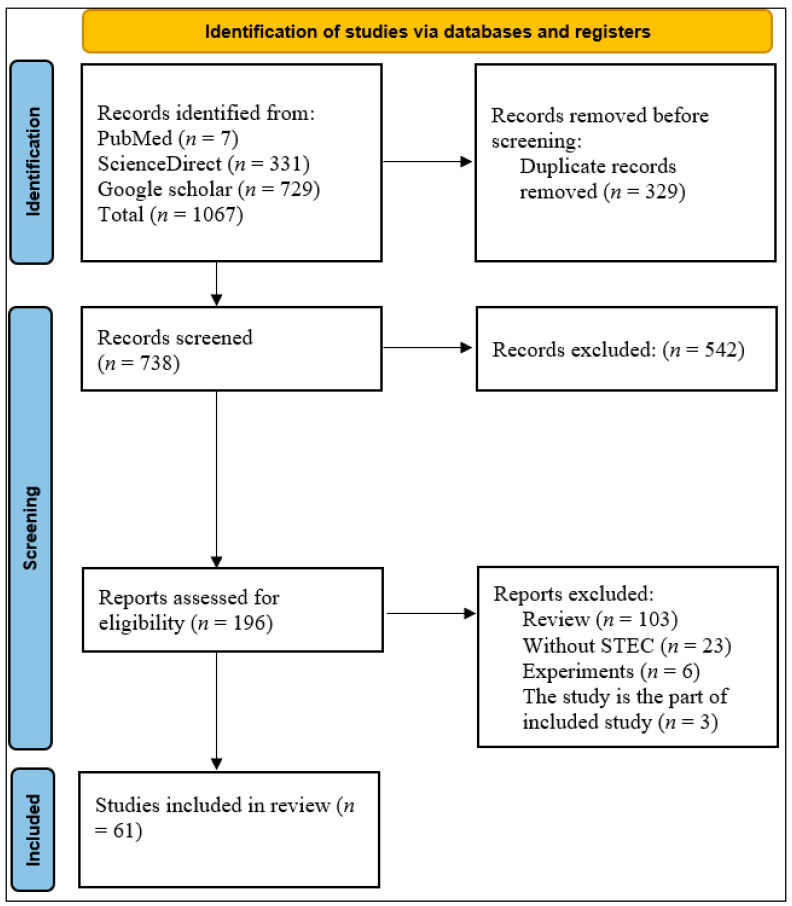
PRISMA flow chart representing selection of studies for inclusion in systematic review of antibiotic resistance and Shiga toxin-producing *Escherichia coli* in chickens.

A total of 1067 articles were retrieved from databases. After removing 329 duplicate articles, approximately 738 articles remained. Another 542 articles were excluded after screening the titles, abstracts, and aims of the studies. Finally, 196 full-length articles were thoroughly reviewed using predetermined selection criteria, and 61 studies were included in this meta-analysis (Figure 1). The Joanna Briggs Institute (JBI) Critical Review Quality Assessment score ranges from 1 to 9. All 61 studies included in our analysis received a score of five or higher.

**Figure 2:**
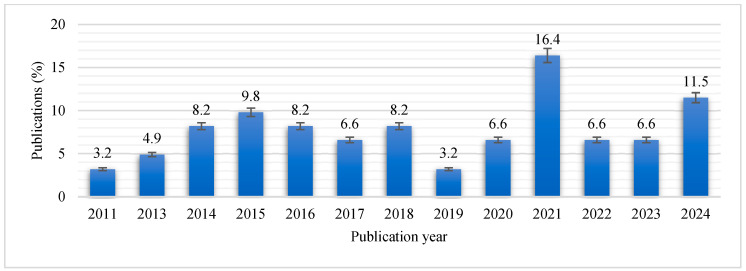
Number of publications included in this study from 2011 to 2024.

Figure 2 shows the distribution of the included articles by year of publication. There were no publications from 2012. The year 2021 had the highest (*n* = 10; 16.4%) number of publications, and 2011 (*n* = 2; 3.2%) had the lowest number of publications.

### 2.2. Study Characteristics

The number of Shiga toxin-producing *E. coli* in chicken isolates per study ranged from 1 to 444, including 385 (*stx1*), 364 (*stx2*), and 940 (STEC), with all the studies published between 2011 and 2024. Antibiotic resistance data were only extracted from studies that specifically investigated antibiotic susceptibility. A summary of the 61 studies that were part of our final meta-analysis can be found in Table 1.

**Figure 3:**
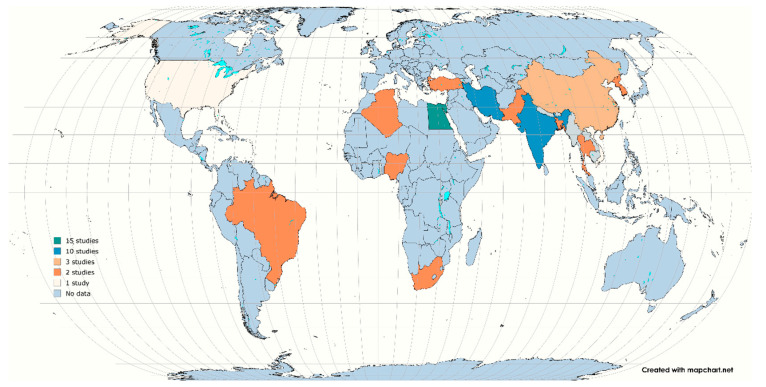
World map showing number of articles from different countries which reported STEC in chicken (https://www.mapchart.net/world.html (accessed on 17 April 2025)). The blue color signifies the lakes.

Based on the separation of countries by continent (United Nations Association) (https://simple.wikipedia.org/wiki/List_of_countries_by_continents (accessed on 5 February 2025)), Asia recorded the highest number of studies (*n* = 36, 59%), followed by Africa (*n* = 21, 34.4%), North America (*n* = 2, 3.8%), South America (*n* = 1, 1.6%), and mixed countries (*n* = 1, 1.6%). Most studies were conducted in Egypt (*n* = 15); India and Iran (*n* = 10 each); China (*n* = 3); Korea, Turkey, Brazil, South Africa, Algeria, Nigeria, Pakistan, Bangladesh, and Thailand (*n* each = 2); and USA, Vietnam, Tunisia, and Switzerland and Germany (*n* = 1 each) (Figure 3). The eligible studies predominantly reported on the prevalence of STEC isolates in meat, eggs, cloacal swabs, GIT content, intestines, faeces, carcasses, chicken sandwiches, and caecum. Only studies published in English that focused on STEC, and antibiotic resistance were included.

### 2.3. Results of Meta-Analysis of Overall Prevalence

**Figure 4:**
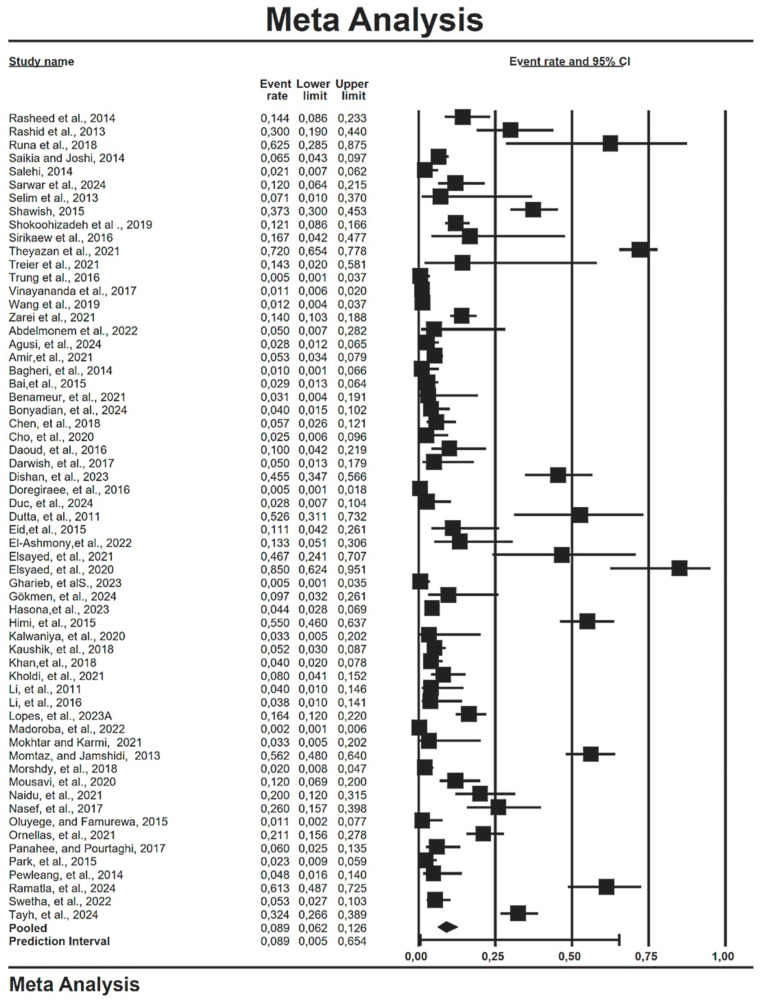
Forest plot showing estimated pooled global prevalence of Shiga toxin-producing *E. coli* [18,19,20,21,22,23,24,25,26,27,28,29,30,31,32,33,34,35,36,37,38,39,40,41,42,43,44,45,46,47,48,49,50,51,52,53,54,55,56,57,58,59,60,61,62,63,64,65,66,67,68,69,70,71,72,73,74,75,76].

High heterogeneity was observed in the studies examining the prevalence of Shiga toxin-producing *E. coli*, influenced by factors such as the overall samples and geographic regions. In this systematic review and meta-analysis, random effects meta-analyses were employed to estimate the prevalence of STEC. The global pooled prevalence estimate (PPE) of STEC was found to be 8.9% (0.890; 95% CI: 0.620–0.126; df = 60; I^2^ = 95%; *p* = 0.001) (Table 2 and Figure 4). According to Table 2, *stx1* had the highest PPE of 12.9% (0.129; 95% CI: 0.081–0.199; df = 33; I^2^ = 92%; *p* = 0.001), followed by *stx2* with 11.8% (0.118; 95% CI: 0.077–0.176; df = 36; I^2^ = 91%; *p* = 0.001).

### 2.4. Subgroup Analyses

#### 2.4.1. Subgroup Analysis of Shiga Toxin-Producing *E. coli*

Visceral organs registered the highest PPE at 36.7% (95% CI: 0.060–0.983; Q = 153.763; df = 3; I^2^ = 98.0%; *p* = 0.001) from four studies, with 220 being STEC-positive, followed by six studies using faecal samples with a PPE of 25.6% (95% CI: 0.079–0.578; Q = 58.324; df = 5; I^2^ = 91.4%; *p* = 0.001), while the lowest was observed in meat, with a PPE of 9.7% (95% CI: 0.058–0.158; Q = 515.654; df = 34; I^2^ = 93.4%; *p* = 0.001) from thirty-five studies. According to the study year, a subgroup analysis revealed that the highest PPE for STEC in chicken appeared in the 2021 to 2024 period, at 9.2% (95% CI: 0.054–0.251; Q = 583.373; df = 24; I^2^ = 95.9%; *p* =0.001), followed by the years 2011–2020, with a PPE of 6.9% (95% CI: 0.044–0.108; Q = 581.243; df = 35; I^2^ = 93.9%; *p* = 0.001).

Three microbiological diagnostic techniques were used to identify STEC, and the multiplex polymerase chain reaction (m-PCR) technique showed the highest detection sensitivity with a PPE of 21.0% (95% CI: 0.088–0.420; Q = 31.846; df = 4; I^2^ = 87.4%; *p* = 0.001). This was followed by the PCR technique, with a PPE of 17.6% (95% CI: 0.119–0.252; Q = 921.522; df = 51; I^2^ = 94.5%; *p* = 0.001) from fifty-two studies. The CHROMagar and MALDI-TOF-MS techniques were not included in the meta-analysis due to the low number of studies using them.

Our analyses showed that Egypt had the highest PPE at 26.4% (95% CI: 0.101–0.533; Q = 238.745; df = 14; I^2^ = 94.1%; *p* = 0.001) from fifteen studies, while the lowest was observed in China, with a PPE of 3.3% (95% CI: 0.018–0.060; Q = 0.214; df = 2; I^2^ = 0%; *p* = 0.001) from three studies, as shown in Table 2. Asia had the highest PPE of 15.6% (95% CI: 0.093–0.248; Q = 696.504; df = 35; I^2^ = 94.9%; *p* = 0.001), compared to Africa with a PPE of 15.5% (95% CI: 0.071–0.305; Q = 330.906; df = 20; I^2^ = 93.9%; *p* = 0.001), while South America and North America were not included in the meta-analysis due to the low number of studies conducted on each continent.

With regard to virulence genes, the *hlyA* gene had a comparatively higher PPE at 22.6% (95% CI: 0.126–0.370; Q = 11.671; df = 4; I^2^ = 65.7%; *p* = 0.001) as compared to the *eaeA* gene at 14.8% (95% CI: 0.087–0.241; Q = 193.306; df = 17; I^2^ = 91.2%; *p* = 0.001), while the *exhA* gene was not included in the meta-analysis due to its appearance in a low number of studies. With respect to serotypes, our analyses showed that O157 had the highest PPE of 80.5% (95% CI: 0.520–0.940; Q = 2.656; df = 3; I^2^ = 0%; *p* = 0.001), while O111 had the lowest PPE of 3.8% (95% CI: 0.081–0.079; Q = 6.258; df = 4; I^2^ = 35.9%; *p* = 0.001) (Table 2).

Meta-regression was conducted to assess the influence of various covariates as moderators of cumulative prevalence. The moderators included factors such as samples, countries, methods, and years. The analysis showed that all the covariates had an *R^2^* of 0.00% influence on the prevalence of *E. coli* isolates from chickens (Table 3).

#### 2.4.2. Antibiotic-Resistant STEC Subgroup Analysis

A random effects model was used to examine seven antibiotic subgroups from three or more studies to determine the prevalence of antibiotic-resistant STEC. As a result, ampicillin had the highest PPE at 28.8% (0.288; 95% CI: 0.145–0.490; df = 10; I^2^ = 96%; *p* = 0.0001), followed by tetracycline with a PPE of 25.2% (0.252; 95% CI: 0.119–0.457; df = 9; I^2^ = 97%; *p* = 0.001) and neomycin with 23.3% (0.233; 95% CI: 0.059–0.596; df = 2; I^2^ = 80%; *p* = 0.001), while Amoxicillin had the lowest PPE of 3.0% (0.030; 95% CI: 0.006–0.134; df = 4; I^2^ = 79%; *p* = 0.001), as shown in Table 4.

## 3. Discussion

Herein, we conducted an in-depth study to investigate the prevalence of antibiotic resistance and STEC in chickens. A total of 61 peer-reviewed articles published between 1 January 2011 and 25 November 2024 were used. Most of the published articles came from Asia and Africa. To the best of our knowledge, this is the first meta-analysis of the global prevalence of antibiotic resistance and STEC in chickens. With these data, we hope to gain a better understanding of the prevalence and resistance patterns of STEC in different parts of the world and thus contribute to preventing the spread of STEC resistance in chickens.

Based on the meta-analysis results in this study, the overall prevalence of STEC in chickens was estimated to be 8.9%, with estimates ranging from 1.1% to 72.2%. Our findings are consistent with a study conducted in 2019 by Alizade et al. [77] which looked at the STEC detection rate in Iran from 1990 to 2017 and estimated the combined prevalence at 9%. However, our results are higher than those obtained in China, where the prevalence is 6% [14]. These differences might stem from differences in the sample size, methodology used for detection, and number of studies included in the current study. One important pathogen that can lead to foodborne illnesses and presents a major public health concern is STEC [14]. The close interconnection between farming and household environments creates an ideal setting for the exchange of genetic material between human and poultry *E. coli* through horizontal gene transfer [11].

The *stx1* toxin had the higher PPE of 12.9% compared with 11.8% for *stx2*. The *stx* family is a family of cytotoxic proteins that have a pentamer of B subunits (7.7 kDa apiece) that facilitate binding to particular receptor molecules, and an A subunit (around 32 kDa) with N-glycosidase activity that is noncovalently attached [1,78]. The strains’ ability to cause serious infections in humans or animals varies, as does the type or amount of STEC produced [1]. Shiga toxins are known to cause severe gastroenteritis and are critical for STEC virulence in humans [7]. Numerous outbreaks of bacterial foodborne diseases have been linked to consumption of meat, whether cooked or not, contaminated with STEC strains [7,33]. The pathogenic forms of *E. coli* are significant as food and waterborne pathogens since the faecal–oral route is the usual way that *E. coli* spreads from animals to humans [79,80].

Samples used in this meta-analysis were classified as meat, cloacal swabs, faeces, visceral organs, and eggs. The subgroup analysis at the sample level showed that visceral organs registered the highest PPE at 36.7%. Our study witnessed a 60.0% decrease globally in the PPE of STEC in chicken during the period of 2017–2019. However, periodic analysis revealed a 10.7% initial increase between the 2017–2019 and 2020–2022 intervals, which was followed by a continuous decrease of 8.3% during the 2023–2024 interval. The failure of animal disease management programmes around the world and the use of increasingly sophisticated diagnostic methods over time may be the cause of the ongoing rise in STEC infection in chickens.

The number and quality of studies have increased in recent years as a result of the use of the latest diagnostic tools, such as molecular methods, particularly PCR [81,82]. We conducted a meta-analysis based on the numerous diagnostic techniques used, such as PCR, m-PCR, CHROMagar STEC, and MALDI-TOF-MS. In this meta-analysis, m-PCR had a high PPE of 21.0%, followed by PCR with a PPE of 17.6%. It has also been discovered that molecular techniques like PCR are more successful than conventional culture-based techniques for detecting bacterial infections [82,83]. The chromogenic medium CHROMagar provides advantages in cost-effectiveness and simplicity when compared to traditional tests, such as API systems and Vitek 2 ID systems. Nonetheless, it requires more time than molecular assays such as PCR [84]. Kalule et al. [85] indicated that their proprietary real-time PCR assay served as a dependable alternative to traditional diagnostic methods, providing enhanced sensitivity and specificity. Nonetheless, CHROMagar STEC detection is limited to a supplementary function because of its elevated false positivity rates and should be employed alongside more precise techniques such as real-time PCR.

This study confirmed the prevalence of STEC serogroups associated with human diseases such as O157, O103, O26, O111, and O145. Our results revealed that O157 had the highest PPE of 80.5%. One of the most well-known STECs, *E. coli* O157, has been linked to numerous foodborne illnesses globally [86]. Of the non-O157 STEC serogroups, 71% of infections caused by STEC are attributed to the serogroups O26, O45, O103, O111, O121, and O145 [87]. The primary cause of haemolytic uraemic syndrome (HUS) is *E. coli* O157:H7; additional STEC serotypes have been linked to serious illness and widespread outbreaks [88]. A high PPE of 22.6% for the Alpha-hemolysin (*HlyA*) gene was found in this meta-analysis. The current study’s analysed published articles indicate that STEC has been detected in chickens in different countries globally. Egypt, an African country, showed the highest PPE of 26.4%, followed by India, in Asia, with a PPE of 20.2%. Other countries, including Korea, Türkiye, Brazil, South Africa, USA, Algeria, Nigeria, Pakistan, Vietnam, Bangladesh, Thailand, Tunisia, and Switzerland and Germany were not included in the meta-analysis due to their low number of studies.

Antibiotic-resistant strains of *E. coli* are a major public health concern as they may spread to humans via food chains or direct human contact with infected birds [89]. Their increasing trend may be attributed to the widespread use of antibiotics in animal husbandry for both prophylactic and growth-promoting purposes. Occasionally, farmers disregard the recommended dosages and withdrawal periods, leading to antibiotic resistance [90]. Ampicillin is a semi-synthetic *β*-lactam antibiotic, commonly used to treat *E. coli* infections in both humans and livestock; however, there has been a recent increase in its resistance rate [91].

This systematic review and meta-analysis showed that antibiotic-resistant STEC in chickens was isolated from various sample types such as meat, cloacal swabs, faeces, visceral organs, eggs, and mixed samples. The meta-analyses showed that the PPE of antibiotic resistance by STEC was higher against ampicillin (28.8%). This is lower than what was observed in previous studies conducted in Spain, South Africa, and Flagstaff (60%, 58.3%, and 51%, respectively) [92,93,94]. These differences may be elucidated by the divergent drug administration policies of the various countries. It has also been speculated that the increased frequency of multidrug-resistant strains is due to repeated exposure to antibiotics in native agricultural strains of *E. coli*.

Antibiotics such as azithromycin, fosfomycin, and meropenem are now used and recommended to treat early-stage STEC infections, especially those caused by *E. coli* O157:H7. These medications are said to be effective in inhibiting the release of Shiga toxin and preventing kidney failure, all while eradicating the pathogen [11]. Analysis of the included studies revealed the PPEs of tetracycline and ampicillin to be 25.2% and 28.8%, respectively. A study conducted by Buvens et al. [95] discovered that intimin-positive, non-O157 STEC strains exhibited greater resistance to tetracycline, kanamycin, and streptomycin. Multidrug-resistant APEC strains have been detected in wild birds, posing a risk of transmission to humans and commercial poultry through mechanical vectors [96]. In the United States, it is currently not recommended to treat STEC infections in humans with antibiotics because some research indicates that doing so could make the condition worse by causing the temperate phage that carries the Shiga toxin (*Stx*) genes to enter the lytic cycle and worsen the tissue damage and symptoms that patients experience from the toxin [11,97]. In this study, the public health implications of multidrug-resistant isolates were observed in four studies.

The World Health Organization (WHO) states that a “One Health” approach is especially relevant in the following areas of work: environmental health, food safety, zoonotic disease control, laboratory services, neglected tropical diseases, and antimicrobial resistance (https://www.emro.who.int/international-health-regulations/areas-of-work/one-health.html, accessed on 13 April 2025). The presence of STEC in chickens is a global concern, as this pathogen is a leading cause of enteric diseases, including diarrhoea and haemolytic uremic syndrome, and its zoonotic isolates pose a risk to human health [98]. The global demand for poultry has led to a shift toward intensive farming practices, which increases the risk of infection transmission, including zoonoses, and affects animal health and productivity [99,100,101]. The prolonged use of antibiotics in animal production has led to the emergence of antimicrobial resistance (AMR) in microbial reservoirs, which poses a significant threat to livestock, particularly poultry [100,101].

## 4. Materials and Methods

### 4.1. The Design of the Study

This systematic review and meta-analysis followed the Preferred Reporting Items for Systematic Reviews and Meta-Analyses (PRISMA) guidelines [16,88]. Inclusion and exclusion criteria were defined with regard to the relevance of the references in order to achieve the study goals.

### 4.2. Ethics

Ethical approval was not necessary for this meta-analysis because it used data and information from publicly available published literature.

### 4.3. Review Question

The review question was as follows: what are the global prevalences of antibiotic resistance and Shiga toxin-producing *Escherichia coli* in chickens?

### 4.4. Search Strategy

A thorough, comprehensive, systematic search of electronic databases was conducted over 5–20 July 2024, and one reviewer (T.R.) updated the search on 25 November 2024. The search included electronic databases (PubMed/Medline, ScienceDirect, Scopus, Google Scholar). Eligible studies published between January 2011 and 25 November 2024 were considered for inclusion. The search strategy was not limited by language. In accordance with Medical Subject Heading (MeSH), all descriptors used in databases were defined. The searching strategy utilised combinations of keywords such as “prevalence” OR “incidence”, AND “Shiga toxin-producing *Escherichia coli*” OR “Shiga toxin-producing *E. coli*”, OR “STEC” OR “Shiga” AND “antibiotic resistance” OR “antibiotic susceptibility” AND “chickens” (“OR” and “AND”) as necessary in advanced database searches. All steps in data extraction were carried out by at least two independent researchers, and inconsistencies were resolved through discussion.

### 4.5. Inclusion and Exclusion Criteria

Full-text publications were screened according to the following inclusion criteria: (1) studies where the full texts were available; (2) studies conducted from 2011 onwards; (3) studies clearly describing sample information; (4) studies written in English; (5) studies investigating the prevalence of antibiotic resistance and Shiga toxin-producing *E. coli* in chickens. Full-text publications were excluded for one or more of the following reasons: (1) sample source not being described or a lack of sample information; (2) being reviews or meta-analyses; (3) not being written in English.

### 4.6. Data Extraction

Data from eligible studies were extracted independently by three reviewers (T.R., G.K., and M.T.) and stored in Microsoft Excel spreadsheets. The details recorded for each study included the first author’s name, year of publication, country or region, sample type, sample size, and number of isolates. Microsoft Excel^®^ (Microsoft Corporation, Redmond, WA, USA) was used to manage the retrieved studies. Duplicates were removed and the titles and abstracts of all the retrieved studies were screened. The full texts of potentially eligible studies were assessed in detail against the inclusion criteria and added to the extraction collection. Any disagreements between reviewers during each phase of screening were resolved by discussion or by involving a third person.

### 4.7. Quality Assessment

The articles’ quality was carefully assessed by three authors. Whether the study satisfied the selection criteria or whether an article’s eligibility was assessed by looking at the full texts of the articles. Methodological validity was assessed for each study design using the Joanna Brigg Institute’s (JBI) quality assessment manual [102]. Studies were assessed using the critical appraisal checklists (Table S1), and articles with an average score between 50% and 75% were considered to be of good quality; items with values above 75% were considered to be of high quality. Good- and high-quality articles were included in this systematic review and meta-analysis. In addition, studies without clear method descriptions or with missing results of interest were not included.

### 4.8. Outcome

The main focus of this study was to determine the global prevalence of antibiotic resistance and Shiga toxin-producing *Escherichia coli* in chickens.

### 4.9. Data Processing and Analysis

A meta-analysis of the prevalence of Shiga toxin-producing *Escherichia coli* in chickens was conducted using the comprehensive meta-analysis software v.4.0 (https://www.meta-analysis.com/) [103,104]. A random effects model was used to calculate the pooled prevalence estimate of antibiotic resistance and Shiga toxin-producing *E. coli* in chickens. The presence of heterogeneity was determined using I^2^ statistics. A value close to 0% indicated no heterogeneity, while values close to 25%, 50%, and 75% indicated increasing heterogeneity. The results were presented in the form of a table and a forest plot. Publication bias was assessed using the Beg and Mazumdar rank correlation test and Egger’s regression test. A *p*-value ≥ 0.05 indicated a lack of publication bias [105]. Because the test (meta-regression) had a low power, 0.25 was deemed significant. The multivariate meta-regression analysis included all components with significant *p*-values.

### 4.10. Test for Publication Bias Due to Small-Study Effects

Statistical tests for funnel plot asymmetry have limitations, and their use is recommended only when a sufficient number of studies (*n* ≥ 10) and low heterogeneity (I^2^ < 50%) are present [104,105,106]. Regrettably, none of the meta-analyses in this study fulfilled these requirements, precluding a reliable assessment of publication bias.

## 5. Limitations

The limitations of our meta-analysis are as follows: (a) It is possible that relevant articles published in languages other than English were not captured by the search strategy, which was limited to English-language publications. (b) Given the limited number of studies conducted in certain countries, the results may not fully represent the entire world. (c) There were more study reports from some continents compared to others. (d) Due to the limited number of investigations, the majority of STEC serotypes were excluded from this meta-analysis. (e) When a high level of heterogeneity is present, accurately assessing the actual results of statistically significant publication bias tests becomes challenging. (f) It is essential for readers to exercise caution when interpreting our pooled analyses and subgroups, given the considerable diversity observed across all studies. (g) Our study protocol was not registered on the standard PROSPERO platform, as is customary for other studies.

## 6. Conclusions

This systematic review and meta-analysis study provides comprehensive information on antibiotic resistance and STEC in chickens over the past 14 years worldwide, which appears to be increasing and spreading. The information provided here is expected to support worldwide epidemiological surveillance on the prevalence and antibiotic resistance of STEC in chickens. STEC carries significant “One Health” implications due to its zoonotic nature, presenting risks to both human and animal health. The state of STEC antibiotic resistance in chickens is still concerning and poses a major threat to public health. The results of this study will help shape prevention and control strategies against antibiotic-resistant STEC in chickens. Furthermore, to combat AR in chickens, strengthened control practices and a “One Health” collaborative research approach are necessary.

## Figures and Tables

**Table 1:** Description of eligible published research articles from January 2014 to November 2024 included in study.

No.	Citation	Country	Samples	Diagnostic Methods	Antibiotic Methods	Total No.	No. of Isolates	*stx1*	*stx2*	STEC
1	Rasheed et al. [18]	India	Meat and eggs	Culture media and PCR	−	90	45	13	11	13
2	Rashid et al. [19]	India	Meat	Culture media, serology, and PCR	−	50	20	9	5	15
3	Runa et al. [20]	Bangladesh	Cloacal swabs	Culture media, serology, and PCR	−	8	5	−	−	5
4	Saiki and Joshi, [21]	India	Meat	Culture media and PCR	−	336	22	22	0	22
5	Salehi [22]	Iran	GIT content	Culture media and PCR	−	145	290	0	3	−
6	Sarwar et al. [23]	Pakistan	Multiple	MALDI-TOF, VAGs, and PCR	DD	75	29	6	7	9
7	Selim et al. [24]	Egypt	Meat	Culture media and PCR	−	14	3	1	−	−
8	Shawish [25]	Egypt	Meat	Culture media, serology, and PCR	−	150	57	14	14	56
9	Shokoohizadeh et al. [26]	Iran	Meat	Culture media, serology, and PCR	DD	257	93	−	−	31
10	Sirikaew et al. [27]	Thailand	Meat	Culture media, serology, and PCR	−	12	2	−	−	2
11	Theyazan et al. [28]	Egypt	Intestines	CHROMagar STEC	−	200	158	8	25	144
12	Treier et al. [29]	Switzerland and Germany	Meat	Brolacin STEC agar or CHROMagar	−	7	3	1	1	1
13	Trung et al. [30]	Vietnam	Faeces	Culture media and PCR	−	188	1	1	−	−
14	Vinayananda et al. [31]	India	Eggs	Culture media, serology, and PCR	−	840	239	−	−	9
15	Wang et al. [32]	Algeria	Meat	Culture media, serology, and PCR	−	248	141	−	3	−
16	Zarei et al. [33]	Iran	Meat	Culture media, serology, and PCR	DD	257	93	15	31	36
17	Abdelmonem et al. [34]	Egypt	Meat	PCR	−	20	1	−	1	1
18	Agusi et al. [35]	Nigeria	Cloacal swabs	PCR	−	179	178		5	
19	Amir et al. [36]	Pakistan	Faeces and meat	PCR	DD	400		19	18	21
20	Bagheri et al. [37]	Iran	Carcasses	PCR	−	102	204		1	
21	Bai et al. [38]	China	Meat	CHROMagar STEC	−	205	−	−	−	6
22	Benameur et al. [39]	Algeria	Faeces	MALDI-TOF-MS	DD	32	−	−	1	−
23	Bonyadian et al. [40]	Iran	Meat	Not specified	−	100	84	−	1	4
24	Chen et al. [41]	USA	Meat	PCR	−	105	25	−	−	6
25	Cho et al. [42]	Korea	Meat	CHROMagar STEC	−	133	79	1	1	2
26	Daoud et al. [43]	Luxor city	Meat	PCR	−	50	6	2	5	
27	Darwish et al. [44]	Egypt	Meat	PCR	−	40		2	2	
28	Dishan et al. [45]	Türkiye	Meat	PCR	DD	100	77	24	23	35
29	Doregiraee et al. [46]	Iran	Cloacal swabs	PCR	−	500	444	−	2	−
30	Duc et al. [47]	Vietnam	Meat	PCR	−	72	7	−	−	2
31	Dutta et al. [48]	India	Faeces	m-PCR	−	19	42	8	6	10
32	Eid et al. [49]	Egypt.	Eggs	PCR	−	200	36	−	4	
33	El-Ashmony et al. [50]	Egypt	Meat	VITEK2/PCR	−	−	30	2	3	4
34	Elsayed et al. [51]	Egypt	Faecal swabs	PCR	−	15	7	6	6	7
35	Elsyaed et al. [52]	Egypt	Faeces	PCR	−	20	17	17	15	−
36	Gharieb et al. [53]	Egypt	Visceral organs	PCR	−	200	110	1	−	−
37	Gökmen et al. [54]	Turkey	Meat	m-PCR	−	31	−	3	−	−
38	Hasona et al. [55]	Egypt	Cloacal swabs and internal organs	PCR	−	410	29	5	5	18
39	Himi et al. [56]	Bangladesh	Cloacal swabs and eggs	PCR	−	120	66	−	66	66
40	Kalwaniya et al. [57]	India	Meat	PCR	−	30	−	1	−	−
41	Kaushik et al. [58]	India	Meat and eggs	m-PCR	−	252	62	−	13	−
42	Khan et al. [59]	India	Meat	PCR	DD	200	34	−	−	8
43	Kholdi et al. [60]	Iran	Meat	PCR	−	100	−	−	−	8
44	Li et al. [61]	China	Meat	PCR	−	50	−	−	−	2
45	Li et al. [62]	China	Meat	PCR	−		52	1	−	2
46	Lopes et al. [63]	Brazil	Cloacae and carcases	PCR	DD	213	−	−	−	35
47	Madoroba et al. [64]	South Africa	Meat	PCR	−	1758	−	−	−	4
48	Mokhtar and Karmi [65]	Egypt	Meat	PCR	−	30	−	−	−	1
49	Momtaz and Jamshidi [1]	Iran	Meat	PCR	DD	422	146	80	5	82
50	Morshdy et al. [66]	Egypt	Chicken sandwiches	m-PCR	−	250	73	5	2	−
51	Mousavi et al. [67]	Iran	Meat	PCR	−	100	−	12	4	−
52	Naidu et al. [68]	India	Multiple	PCR	−	65	13	3		−
53	Nasef et al. [69]	Egypt	Multiple	PCR	−	50	20	9	13	−
54	Oluyege and Famurewa [70]	Nigeria	Faeces	PCR	−	293	87	1	−	−
55	Ornellas et al. [71]	Brazil	Carcasses and cloacae	PCR	DD	−	171	−	−	36
56	Panahee and Pourtaghi [72]	Iran	Meat	PCR	−	84	9	−	5	−
57	Park et al. [73]	Korea	Meat	PCR	−	233	176	−	−	4
58	Pewleang et al. [74]	Thailand	Meat	PCR	−	62	−	3	1	−
59	Ramatla et al. [7]	South Africa	Faeces	PCR	−	480	62	29	38	−
60	Swetha et al. [75]	India	Meat	PCR	−	150	16		8	8
61	Tayh et al. [76]	Tunisia	Caecum	PCR	−		222	61	10	72

Disc diffusion (DD), polymerase chain reaction (PCR), multiplex PCR (m-PCR), Shiga toxin-producing *E. coli* (STEC).

**Table 2:** A summary of the meta-analysis of the prevalence of STEC in chicken.

Risk Factors	Number of Studies	Pooled Estimates			Measure of Heterogeneity				Publication Bias
		Sample Size	STEC-Positive	I^2^ (95%CI)	Q Value	I^2^	*Q*	Tau^2^	*p*-Value (*p* < 0.05)
**Overall**									
STEC	61	9973	940	8.9% (6.2–12.6)	1299.759	95.4	<0.001	2.158	0.353
*stx1*	34	2874	385	12.9% (8.1–19.9)	440.987	92.5	<0.001	1.969	0.116
*stx2*	37	3281	364	11.8% (7.7–17.6)	286.650	90.7	<0.001	1.765	0.107
**Samples**									
Meat	35	4415	379	9.7% (5.8–15.8)	515.654	93.4	<0.001	2.456	0.754
Cloacal swabs	4	659	30	12.2% (0.8–69.4)	85.235	96.5	<0.001	7.790	0.496
Faeces	6	359	74	25.6% (7.9–57.8)	58.324	91.4	<0.001	2.555	0.188
Visceral organs	4	630	220	36.7% (0.6–98.3)	153.763	98.0	<0.001	21.009	0.500
Mix samples	10	798	203	26.6% (16.8–39.3)	84.168	90.5	<0.001	0.688	0.835
Eggs	2	275	13	−	−	−	−		−
**Virulence genes**									
*eaeA*	18	1257	227	14.8% (8.7–24.1)	193.306	91.2	<0.001	1.409	0.471
*HlyA*	5	185	45	22.6% (12.6–37.0)	11.671	65.7	<0.001	0.383	0.141
*exhA*	2	246	43	−	−	−	−		−
**Serotypes**									
O157	4	580	18	80.5% (52.0–94.0)	2.656	0.00	<0.001	2.656	0.497
O103	3	77	6	12.3% (2.1–47.3)	7.880	74.6	<0.001	2.003	0.117
O26	7	143	22	5.1% (2.8–9.1)	11.413	47.4	<0.001	0.322	0.880
O111	5	186	14	3.8% (1.8–7.9)	6.258	35.9	<0.001	0.258	0.624
O145	3	106	5	4.9% (2.1–11.3)	0.480	0.00	<0.001	0.000	0.601
**Methods**									
PCR	52	6437	882	17.6% (11.9–25.2)	921.522	94.5	<0.001	1.551	0.956
m-PCR	5	237	49	21.0% (8.8–42.0)	31.846	87.4	<0.001	1.131	0.624
CHROMagar STEC	2	284	8	−	−	−	−	−	−
MALDI-TOF-MS	2	107	10	−	−	−	−	−	−
**Years**									
2011–2016	21	3293	301	6.6% (3.6–11.7)	346.308	94.2	<0.001	1.823	0.277
2017–2019	11	2334	100	6.0% (3.0–11.7)	106.741	90.6		1.309	0.937
2020–2022	18	3518	317	10.7% (5.0–21.6)	447.748	96.2		2.831	0.704
2023–2024	11	2082	222	8.3% (4.4–15.1)	170.819	94.1	<0.001	1.113	0.311
**Country**									
India	10	889	112	20.2% (10.8–34.6)	95.044	90.5	<0.001	1.224	0.089
Egypt	15	702	279	26.4% (10.1–53.3)	238.745	94.1	<0.001	4.760	0.805
Iran	10	1638	184	7.7% (3.0–18.4)	214.427	95.8	<0.001	2.302	0.001
China	3	307	10	3.3% (1.8–6.0)	0.214	0.00	<0.001	0.000	0.117
**Continent**									
Asia	36	3779	605	15.6% (9.3–24.8)	696.504	94.9	<0.001	2.813	0.683
Africa	21	2977	259	15.5% (7.1–30.5)	330.906	93.9	<0.001	3.566	0.856
North America	2	384	71	−	−	−	−		−
South America	1	25	6	−	−	−	−		−
Europe	1	7	3	−	−	−	−		−

STEC = Shiga toxin-producing *E. coli*; PCR = polymerase chain reaction; m-PCR = multiplex polymerase chain reaction; MALDI-TOF-MS = matrix-assisted laser desorption ionisation–time of flight mass spectrometry.

**Table 3:** Univariate and multivariate meta-regression analysis of prevalence of *E. coli* isolates from chickens.

Univariate Analysis			Multivariate Analysis	
Covariates	*R^2^*	*p*-Value	*R*^2^ (%)	*p*-Value
Overall			0.144	**-**
Samples	0.000	0.000		
Countries	0.000	0.000		
Methods	0.000	0.000		
Years	0.144	-		

**Table 4:** Pooled prevalence estimates and 95% CIs of antibiotic resistance of STEC isolates from this study.

Antimicrobial Agents	Number of Studies	Number of Isolates	% Prevalence (95%CI)	I^2^ (95%CI)	Tau^2^	Publication Bias *p*-Value
TET	10	359	25.2% (11.9–45.7)	97	1.977	0.788
CIP	10	59	4.9% (2.1–11.1)	86	1.211	0.620
N	3	65	23.3% (5.9–59.6)	80	1.504	0.601
CAF	3	66	5.3% (0.3–50.9)	97	6.414	0.117
AMP	11	322	28.8% (14.5–49.0)	96	1.954	0.815
GEN	7	109	8.7% (4.8–15.2)	88	0.617	0.024
AML	3	9	3.0% (0.06–13.4)	79	1.527	0.601
AMC	5	58	8.7% (3.1–22.1)	89	1.183	0.815
MDR	4	7	0.7% (0.3–1.5)	00	0.0	0.734

Multidrug-resistant (MDR), Amoxicillin and clavulanic acid (AMC), Chloramphenicol (CAF), Tetracycline (TET), Ciprofloxacin (CIP), Gentamycin (GEN), Ampicillin (AMP), Neomycin (N), Amoxicillin (AML).

## Data Availability

All data are included in the manuscript and the Supplementary Files.

## Supplementary Materials

The following supporting information can be downloaded at: https://www.mdpi.com/article/10.3390/antibiotics14060568/s1. Table S1. Checklist of items to include when reporting a systematic review or meta-analysis
